# Symptom-based classification of Parkinson’s disease using retrospective electronic medical records: A STROBE-compliant cross-sectional study

**DOI:** 10.1097/MD.0000000000050608

**Published:** 2026-09-18

**Authors:** HuiYan Zhao, Ojin Kwon, Jae Young Jang, Gyu Ri Jeon, Ye-Chae Hwang, Eunbyul Cho, Jiyun Cha, Eun Kyoung Ahn, Seong-Uk Park, Jung-Hee Jang

**Affiliations:** a KM Science Research Division, Korea Institute of Oriental Medicine, Daejeon, Republic of Korea; b School of Electrical, Electronics and Communication Engineering, Korea University of Technology and Education (KOREATECH), Cheonan, Republic of Korea; c Department of Korean Medicine Cardiology and Neurology, Graduate School, Kyung Hee University, Seoul, Republic of Korea; d Department of Diagnostics, College of Korean Medicine, Wonkwang University, Iksan, Republic of Korea; e Digital Health Research Division, Korea Institute of Oriental Medicine, Daejeon, Republic of Korea; f Department of Internal Korean Medicine, College of Korean Medicine, Daejeon University, Daejeon, Republic of Korea; g KM Data Division, Korea Institute of Oriental Medicine, Daejeon, Republic of Korea; h Department of Cardiology and Neurology, College of Korean Medicine, Kyung Hee University, Seoul, Republic of Korea; i College of Korean Medicine, Daejeon University, Daejeon, Republic of Korea.

**Keywords:** Parkinson’s disease, prognosis, subtype clustering, syndrome differentiation, traditional medicine

## Abstract

This cross-sectional study of electronic medical records (EMRs) was conducted to investigate symptom patterns associated with Parkinson’s disease (PD) duration and severity and to explore a symptom-based classification of PD. Data from 334 patients with idiopathic PD who visited a single hospital in South Korea between 2011 and 2021, were used to extract a range of PD-associated symptoms and traditional East-Asian medicine (TEAM) symptoms. Frequency analysis was performed to identify key clinical symptoms by PD progression and severity. Patients with moderate disease severity exhibited different symptom patterns across disease-duration categories, including differences in dyskinesia, speech disorder, floating pulse, and yellow tongue coating. Hierarchical clustering based on PD-related and traditional East Asian medicine symptoms identified 5 distinct EMR-based subtypes. EMR subtype 1 was characterized by the mildest disease severity and shortest disease duration, whereas the remaining subtypes showed distinct symptom profiles and a generally greater overall symptom burden. This information may be useful for identifying clinically distinguishable EMR-based PD subtypes and symptom patterns associated with disease duration and severity. These preliminary findings provide a basis for future studies investigating whether such patterns may inform individualized approaches to PD management.

## 1. Introduction

Parkinson’s disease (PD) is a progressive neurodegenerative disorder characterized by the loss of dopaminergic neurons in the substantia nigra pars compacta and the appearance of Lewy bodies.^[1]^ The clinical symptoms of PD develop insidiously; they can be classified according to disease progression over time. Prediagnostic features of PD are tremor, constipation, dizziness, fatigue, and hypotension, while common features at onset are bradykinesia, rigidity, and tremor. However, as the disease progresses, more symptoms develop. Dyskinesia is a common motor complication observed during the course of PD, particularly in patients receiving long-term levodopa therapy. In contrast, L‑DOPA-resistant motor disabilities, including postural instability and gait disturbance (PIGD), are commonly observed in late-stage PD.^[1-3]^ Hence, PD exhibits substantial clinical heterogeneity, with symptom profiles varying across disease stages and individuals.^[2,4]^ Consequently, stage-specific approaches that account for disease progression may be required. Therefore, multiple studies have attempted to classify PD into distinct subtypes using clinical manifestations, biomarker profiles, and disease progression trajectories, reflecting the substantial heterogeneity of PD.^[5-9]^

In traditional East-Asian medicine (TEAM), which is based on holism, PD has been effectively treated with few side effects.^[10,11]^ The TEAM diagnostic method of syndrome differentiation (SD) is a comprehensive analysis process based on 4 TEAM diagnostic criteria, which include observation, listening, questioning, and pulse condition.^[12,13]^ SD enables a generalization of the disease location and nature, as well as a determination of the developmental tendency of a disease at a specific stage and herbal prescription.^[14]^ SD diagnosis in TEAM is used to establish an effective TEAM treatment strategy; however, it is associated with complexity, ambiguity, and subjectivity,^[14,15]^ rather than objectively measurable states.^[16]^ In historical literature, SD records of PD were limited to PD’s specific symptoms, such as tremor and trembling.^[17,18]^ In addition, there is no SD content about PD in textbooks at oriental medicine universities in South Korea and China.^[19,20]^ Additionally, research focusing on the role of SD considering the varied clinical symptoms and long progression of neurodegenerative disease has been limited. To compensate for this, researchers have investigated traditional SD patterns according to the longitudinal progression of Alzheimer disease (AD) to develop a staged and multiply-targeted sequential therapy for this disease^[21]^; this is an example of SD research in neurodegenerative diseases. This report demonstrated that, according to SD, AD proceeded from Shen deficiency to phlegm, stasis, and fire in order. It ultimately worsened to severe toxins, when the symptoms changed from memory impairment to function failure.^[21]^ In contrast, a study examining SD patterns in PD reported frequent SD without considering the progressive stages of the disease. These included kidney and liver yin deficiency, qi and blood deficiency, phlegm heat with wind stirring, blood stasis, and wind stirring.^[22]^ However, all data regarding SD of PD used in the previous study were collected from literature articles.^[22]^ There are also some limitations in treating PD using SD in clinical practice because of the complex pathological changes in PD symptoms over time.^[23]^

Unlike previous PD subtype studies that primarily relied on conventional clinical features, biomarkers,^[24]^ neuroimaging characteristics^[25,26]^, or literature-based SD classifications, the present study utilized real-world EMR data and integrated both PD-related and TEAM symptoms to explore symptom-based subtypes according to disease duration and severity. Furthermore, real-world EMR-based clustering studies incorporating TEAM symptom patterns remain limited in East Asian countries.^[27,28]^Therefore, objective guidelines for SD that consider all major symptoms of PD, as well as the progressive duration for patients with PD, are required. In this study, we used real-world data routinely collected from electronic medical records (EMRs) to analyze the characteristics of the TEAM clinical symptom index and herbal medicine while considering PD progression and severity. In addition, an objective classification based on both PD-associated and TEAM symptoms for individual patients with PD has been investigated using the hierarchical clustering analysis (HCA) technique.^[29]^ This is an unsupervised machine-learning technique widely used in many fields to classify each data object into a specific group called a cluster.^[30]^ Symptom clustering can resolve the association between the variables and help detect a symptom pattern. The present study aimed to explore symptom-based EMR subtypes and their associations with disease duration and severity in PD.

## 2. Materials and methods

### 2.1. Study design and ethics approval

The selected anonymized EMR data of patients were obtained from Kyung Hee University Hospital affiliated with Kyung Hee University in South Korea. This cross-sectional study was approved by the Institutional Review Board (approval number: KHNMCOH-2022-02-006). The need for informed consent was waived due to the retrospective nature of the study and the use of anonymized patient data.

### 2.2. Study population and inclusion/exclusion criteria

We identified all patients with PD who visited the hospital for the main diagnosis between January 2011 and December 2021. Inclusion criteria were inpatients and outpatients who met the diagnostic criteria of idiopathic PD (diagnostic code, G20). Exclusion criteria included patients with Parkinsonism such as multiple-system atrophy, progressive supranuclear palsy, corticobasal degeneration, or diffuse Lewy body disease.

### 2.3. Data extraction and management

A preliminary EMR format was prepared based on the list of clinical symptoms and TEAM symptoms/signs investigated and recorded in the real world while caring for patients with PD in hospitals. The following demographic details were extracted: age, sex, disease information (time of PD diagnosis, Unified Parkinson’s Disease Rating Scale [UPDRS] score), Hoehn and Yahr scale (H&Y), clinical symptoms, TEAM diagnostic information including systemic symptoms, and prescription information of herbal medicines. Two trained clinicians from a Korean medicine hospital, specializing in the care of elderly patients with PD, with more than 3 years of clinical experience, extracted data from the EMR format and exported it to an Excel dataset. Each investigator examined the data at least twice to reduce the risk of bias in data collection. Although formal inter-rater reliability statistics were not calculated, discrepancies identified during the review process were discussed and resolved through consensus.

### 2.4. Data standardization

Records with missing information for the variables included in the analysis were completely excluded from the final dataset using listwise deletion. To preserve the actual clinical representation of the collected data, no statistical imputation procedures were performed to replace the missing values. The final EMR data were selected, classified, and standardized by 3 professional experts, including 2 Korean medicine doctors and 1 Chinese medicine doctor. The coding and classification processes were as follows: First, the principal features were presented using common medical terminologies following TEAM diagnostic guidelines to ensure data objectivity and comprehensiveness.^[20,31]^ Second, some principal features with missing variables were excluded. The frequencies of the variables are shown in Table 1. Third, we finally selected the features to analyze and completed a code comparative table to classify variables for each feature, presented in Table S1, Supplemental Digital Content 1. Subsequently, we coded each variable feature as normal, abnormal, or unknown, assigning them 1, 2, and 3, respectively. These numerical values were assigned solely for data standardization and computational processing during PCA and clustering analyses and were not intended to represent a quantitative or clinical ranking of the categories. The coding scheme was developed through expert consensus based on the characteristics of the symptoms and signs. For variables that could not be classified using the normal–abnormal–unknown framework (e.g., sweating, cold and fever, pulse condition, and tongue condition), symptom-specific coding criteria were developed and applied.

**Table 1:** Frequencies of all variables according to features.

Principal feature/sub-feature		Frequency of variables		
		Normal	Abnormal	Other
Appetite		265	124	8
Bradykinesia		28	352	17
Bitter taste		101	53	243
Cold and fever	Cold		140	45
	Fever		99	
	Cold and fever		113	
Dyskinesia		295	54	48
Digestion		275	115	7
Dry mouth		124	218	55
Feces		101	284	12
Gait disturbance		35	350	12
Postural instability		69	305	23
Pain		14	215	168
Pulse intensity	Feeble		17	326
	Solid		52	
	Feeble and solid		2	
Pulse rate	Slow		23	323
	Rapid		51	
Pulse depth	Floating		44	252
	Sunken		101	
Respiration		199	3	195
Rigidity		114	252	31
Sleep disorder		81	311	5
Speech disorder		0	201	196
Swallowing		0	153	244
Sweating	No sweating		14	27
	Unintentional sweating		268	
	Night sweating		44	
	Unintentional sweating and night sweating		44	
Tremor		85	309	3
Thirsty		157	193	47
Tongue color	Light red		153	119
	Pale		4	
	Red		110	
	Dark red		11	
Tongue size	Large		16	381
Tongue coating color	White		160	191
	Yellow		39	
	White and yellow		7	
Tongue coating volume	Lacking		74	266
	Greasy		49	
	Dry		8	
Urine		0	321	76
Weakness		92	205	100

### 2.5. Assessment of the disease progression-based classification

To analyze the characteristics of PD-associated symptoms or TEAM symptoms/signs according to the PD disease progression-based classification, we arbitrarily divided the progressive duration into 3 periods: the period from 0 to 3 years from PD onset (A subdivision), the period of 3 to 7 years (B subdivision), and the period more than 7 years from PD onset (C subdivision). The 3 progressive stages of PD have been defined as before 3 years (early stage), 3 to 7 years (mild to moderate stage), and more than 7 years.^[32-34]^ These cutoffs were selected to distinguish relatively early, intermediate, and longer disease durations and should not be interpreted as established clinical stages of PD. The H&Y score was used for PD severity classification to differentiate between the mild and moderate stages. Scores ≤ 2.5 were considered the mild PD stage, whereas scores > 2.5 were considered the moderate stage.^[35]^

### 2.6. Clustering method for the symptom-based subtype classification

We applied the HCA method to classify all data points into several subtypes (same as groups). As the number of raw clinical features was very large (29), a dimensionality reduction technique was required to improve the interpretability and reliability of the clustering results. Therefore, principal component analysis (PCA) was used to reduce the dimensionality of the data and its many features.^[36]^ Prior to PCA, the data were standardized to ensure all variables contributed equally. The optimal number of principal components was determined based on the cumulative explained variance ratio, resulting in the extraction of 10 new principal features from the 29 raw features. These features reflected the variance of the raw data distribution and were subsequently used to implement the clustering analysis. For the clustering process, agglomerative hierarchical clustering analysis (HCA) was performed using the complete linkage method. To validate the clustering quality and determine the optimal number of clusters, we visually evaluated the dendrogram structure and assessed the Silhouette score. To compare characteristic PD-associated symptoms and TEAM symptoms/signs according to each subtype while also distinguishing the prominent subtype for each principal feature, we drew radar plots relative to each variable of the same feature; this could be used to investigate the symptoms/signs of the 5 subtype groups.

### 2.7. Statistical analysis

First, multinomial logistic regression was used to examine the changes in symptoms according to the progressive duration. In addition, to investigate the prominent clinical symptom or TEAM syndrome for each subtype, the relative ratio of the frequency of variables belonging to each principal feature for subtype numbers 0, 1, 2, 3, and 4 was calculated. Fisher exact test was used to analyze the frequency relative ratio of clinical symptoms and TEAM syndrome terms. Frequency analysis of herbal medicine was also conducted to estimate the distribution of herbal medicine for each subtype. To manage repeated observations from the same patients, each hospital encounter was treated as an independent observation. This approach was adopted because the clinical presentations and disease severity of patients with multiple visits varied significantly over time, representing distinct disease states rather than static replicates. Analyses were performed using the statistical program SAS®, version 9.4 (SAS Institute, Inc.). A 2-sided test was used in all statistical analyses, while the significance level was set at 5%

## 3. Results

### 3.1. Data sample

In total, 398 cases (PD patient encounters) were identified. The age of PD onset was not reported for 1 case; hence, it was excluded from the statistical analysis. Afterward, 397 cases from 334 PD patient records were included for classification based on their symptoms/signs or PD progressive duration (30 patients visited the hospital more than 2 times). The demographic characteristics of the 397 cases are presented in Table 2.

**Table 2:** General characteristics of cases.

	Total	Mild	Moderate
Men, n (%)	121 (30.5)	68 (38.4)	25 (32.1)
Women, n (%)	276 (69.5)	109 (61.6)	53 (67.9)
Age (yr), mean, SD	66.6 (8.8)	65.2 (8.7)	69.7 (8.6)
Age at onset (yr), mean, SD	62.6 (10.1)	60.9 (9.7)	65.5 (9.4)
Progressive duration (yr) mean, SD	4.1 (3.8)	3.7 (3.7)	4.5 (3.6)
H&Y, mean, SD	2.0 (1.1)	1.4 (0.5)	3.5 (0.7)
UPDRS II, mean, SD	12.4 (8.0)	8.8 (5.2)	19.0 (8.6)
UPDRS III, mean, SD	18.9 (12.8)	13.7 (8.3)	30.6 (14.7)

H&Y = Hoehn and Yahr scale, n = number, SD = standard deviation, UPDRS = Unified Parkinson’s Disease Rating scale.

### 3.2. Detailed analysis according to the disease progression-based classification

#### 3.2.1. General characteristics based on PD progressive duration

The demographic characteristics corresponding to the A (n = 219), B (n = 95), and C (n = 83) subdivisions were investigated. In subdivisions A, B, and C, there were 138 (63.0%), 79 (83.2%), and 59 (71.1%) women, respectively. The oldest age at onset was in subdivision A (65.4 ± 9.8 years). PD severity based on the H&Y scale and UPDRS worsened with a longer progressive duration. The patients’ PD severity was categorized as mild (1–2.5) or moderate (3–5) stage based on the H&Y scale. Patient characteristics according to the progressive duration are summarized in Table S2, Supplemental Digital Content 2.

#### 3.2.2. Clinical characteristics according to PD progression

**Figure 1.**
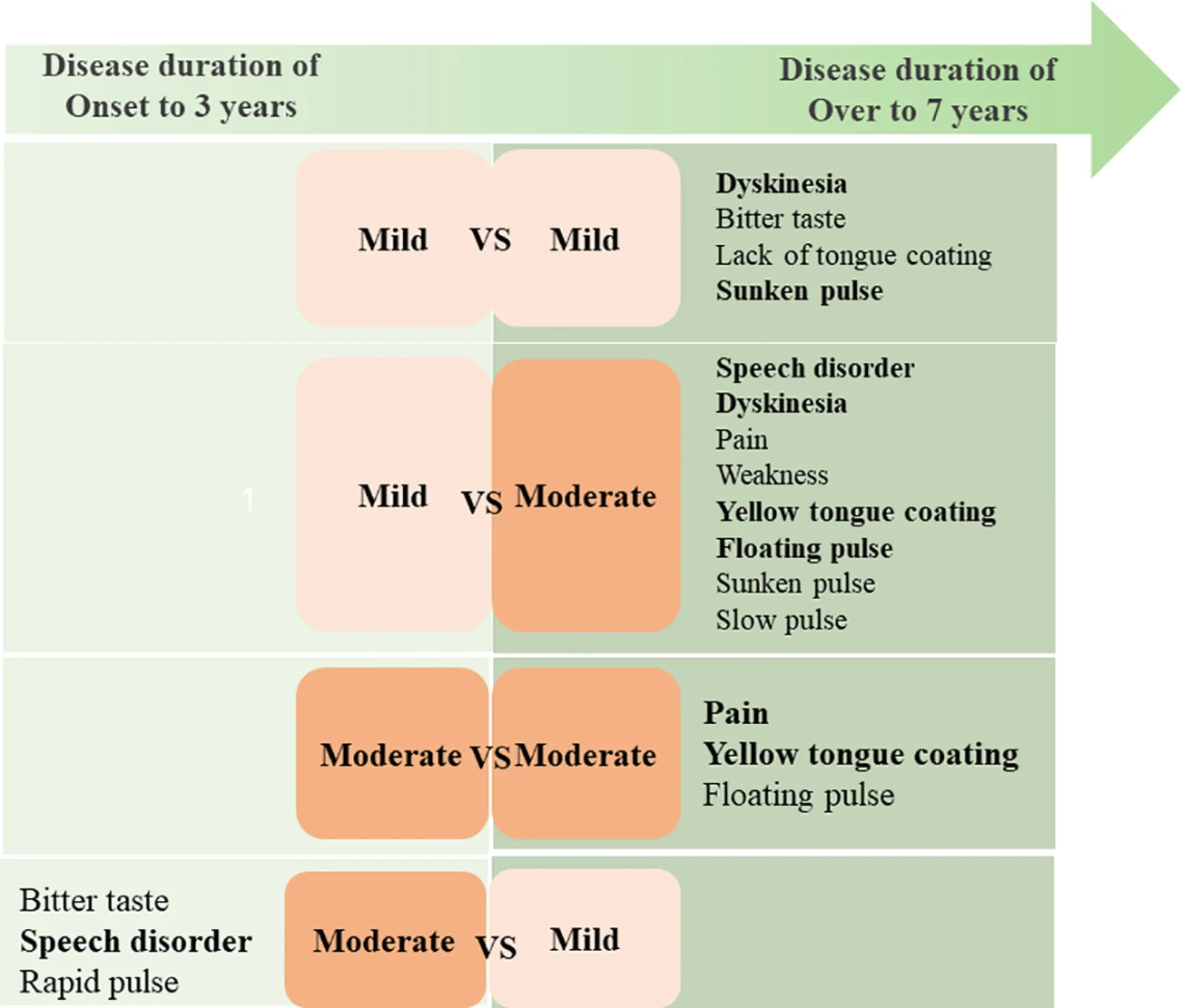
Symptom changes based on disease severity. *Bold text indicates symptoms/signs that showed statistically significant differences between the compared groups (*P* < .05), whereas non-bold text indicates borderline findings (.05 ≤ *P* < .10).

The complete results of the symptom frequency analysis are provided in Table S3, Supplemental Digital Content 3. In the main manuscript, we present symptoms that reached statistical significance (*P* < .05) together with borderline findings (.05 ≤ *P* < .10), which may be of potential clinical interest. We investigated symptom frequencies according to disease severity and progression duration (Table 3 and Fig. 1). Among mild-stage cases, dyskinesia and sunken pulse were significantly more frequent in subdivision C than in subdivision A, whereas bitter taste and lack of tongue coating showed borderline differences. Among moderate-stage cases, pain and yellow tongue coating were significantly more frequent in subdivision C than in subdivision A, whereas floating pulse showed a borderline difference. When mild-stage cases in subdivision A were compared with moderate-stage cases in subdivision C, speech disorder, dyskinesia, yellow tongue coating, and floating pulse were significantly more frequent in the latter group, while pain, weakness, sunken pulse, and slow pulse showed borderline differences. Finally, speech disorder was significantly more frequent in moderate-stage cases in subdivision A than in mild-stage cases in subdivision C, whereas bitter taste and rapid pulse showed borderline differences.

**Table 3:** Frequencies of symptoms or signs according to progressive duration and disease severity.

Parameter	Estimate†	Standard	Wald	*P*-value
		Error	Chi-square	
Subdivision A (mild) vs subdivision C (mild)				
Dyskinesia	−3.2386	0.9445	11.7568	.0006*
Bitter taste	1.8638	1.059	3.0974	.0784
Lack of tongue coating	−0.9123	0.4897	3.4703	.0625
Sunken pulse	−0.9289	0.4534	4.1973	.0405*
Subdivision A (mild) vs subdivision C (moderate)				
Dyskinesia	−3.3928	1.0962	9.5793	.0020*
Floating pulse	−1.6933	0.6607	6.5691	.0104*
Pain	−0.993	0.5241	3.5902	.0581
Slow pulse	−1.4248	0.7843	3.3007	.0692
Speech disorder	−1.5389	0.5469	7.9178	.0049*
Sunken pulse	−1.0647	0.5777	3.3969	.0653
Weakness	−1.8182	1.0621	2.9307	.0869
Yellow tongue coating	−1.4881	0.6775	4.8245	.0281*
Subdivision A (moderate) vs subdivision C (moderate)				
Floating pulse	−1.5041	0.8188	3.3746	.0662
Pain	−1.1658	0.5883	3.9271	.0475*
Yellow tongue coating	−2.3979	1.1599	4.2736	.0387*
Subdivision A (moderate) vs subdivision C (mild)				
Bitter taste	1.9919	1.1257	3.131	.0768
Rapid pulse	1.9459	1.1008	3.1247	.0771
Speech disorder	1.2773	0.5074	6.336	.0118*

Subdivision A: within 3 years of Parkinson’s disease (PD) onset; subdivision B: 3 to 7 years from PD onset; subdivision C: over 7 years from PD onset.

H&Y = Hoehn and Yahr scale, n = number, NR = not reported, PD = Parkinson’s disease, SD = standard deviation, UPDRS = Unified Parkinson’s Disease Rating scale.

† “−” means higher relative rate in subdivision C (each corresponding severity); “+” means higher relative rate in subdivision A (each corresponding severity).

* *P* < .05.

### 3.3. Detailed analysis according to the clustering method of the clinical symptom-based subtype classification

#### 3.3.1. Five subtypes classified based on symptoms using the clustering method

**Figure 2.**
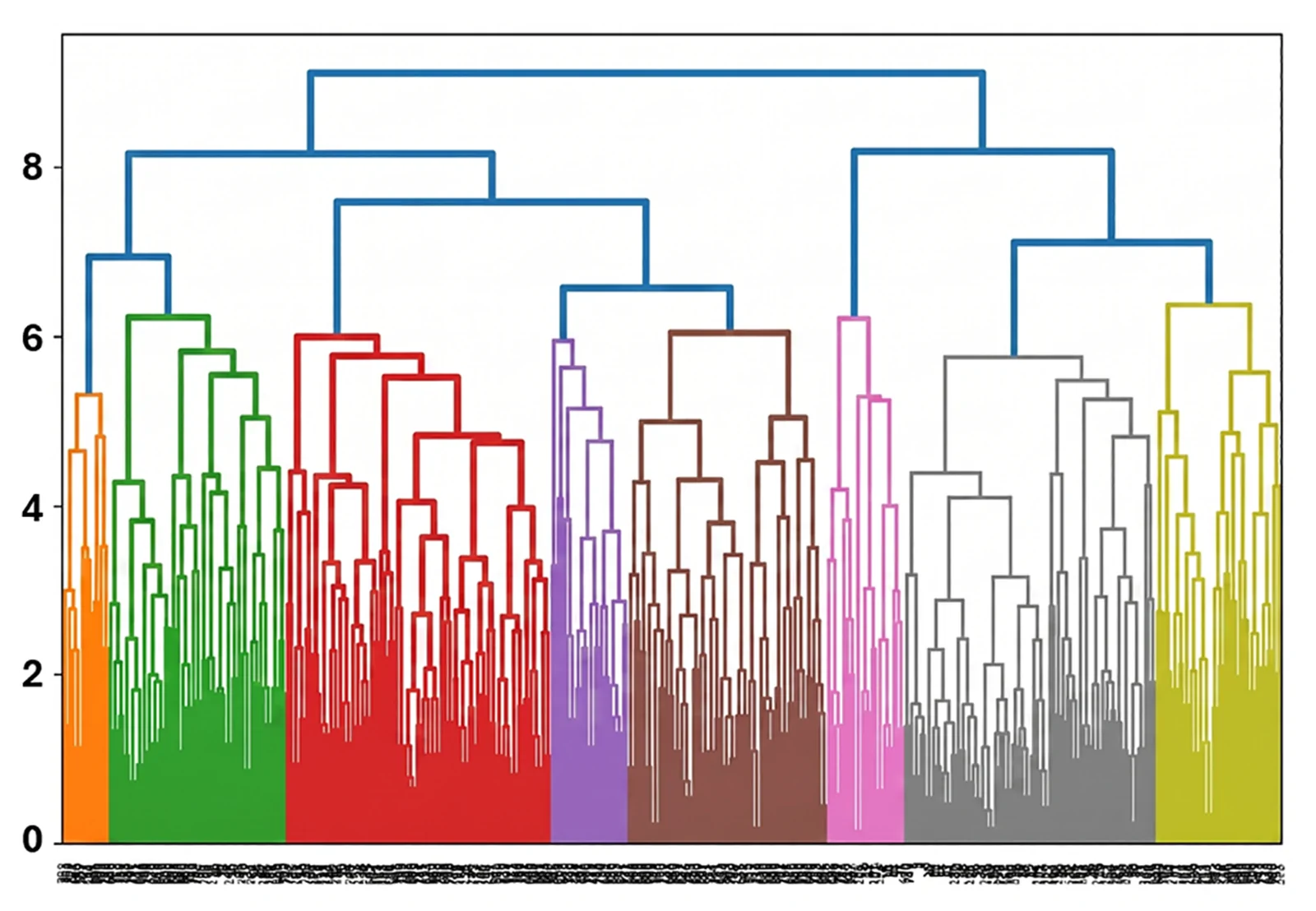
Five dendrograms of hierarchical clustering analysis based on syndrome pattern.

Since there were 29 raw clinical features, 10 new principal clinical features were extracted according to the explained variance ratios in PCA during the dimensionality reduction process. HCA of the symptom-based classification was performed using these 10 principal features, and a dendrogram was created based on the HCA method (Fig. 2). According to the dendrogram after cluster analysis, the optimal number of cluster groups was 5. We ultimately denoted 5 cluster groups as the number of cases in each subtype was evenly distributed in the 5 EMR subtypes: 0 (n = 73), 1 (n = 86), 2 (n = 90), 3 (n = 25), and 4 (n = 123).

#### 3.3.2. Clinical characteristics according to the 5 EMR subtypes

Among 29 raw features, 22 features were distinguished using clustering. These features were significantly different among the 5 EMR subtypes. The results of the relative ratio for symptom proportions by the 5 EMR subtypes are presented in Table 4. The prominent variables for each subtype, identified using radar plots, are demonstrated in Figure S1, Supplemental Digital Content 4 and highlighted in red in Table 4. The distinguishing symptoms for each subtype are as follows (Table 4 and Fig. S1, Supplemental Digital Content 4):

**Table 4:** Pairwise comparisons of the relative ratios for symptoms and signs according to EMR subtype.

Clinical symptom	EMR Subtype 0 (n = 73)	EMR Subtype 1 (n = 86)	EMR Subtype 2 (n = 90)	EMR Subtype 3 (n = 25)	EMR Subtype 4 (n = 123)	*P*-value*
Appetite, n, (%)						.0484*
Normal	45 (61.6)	62 (72.1)	55 (61.1)	14 (56.0)	89 (72.4)	
Abnormal	25 (34.2)	24 (27.9)	35 (38.9)	9 (36.0)	31 (25.2)	
Other	3 (4.1)	0 (0.0)	0 (0.0)	2 (8.0)	3 (2.4)	
Bitter taste in mouth, n, (%)						.0010*
Normal	19 (26.0)	26 (30.2)	8 (8.9)	4 (16.0)	44 (35.8)	
Abnormal	11 (15.1)	11 (12.8)	13 (14.4)	1 (4.0)	17 (13.8)	
Other	43 (58.9)	49 (57.0)	69 (76.7)	20 (80.0)	62 (50.4)	
Bradykinesia, n, (%)						.0054*
Normal	4 (5.5)	8 (9.3)	8 (8.9)	1 (4.0)	7 (5.7)	
Abnormal	67 (91.8)	77 (89.5)	76 (84.4)	19 (76.0)	113 (91.9)	
Other	2 (2.7)	1 (1.2)	6 (6.7)	5 (20.0)	3 (2.4)	
Cold and fever, n, (%)						<.0001*
Cold	28 (38.4)	44 (51.2)	16 (17.8)	4 (16.0)	48 (39.0)	
Fever	14 (19.2)	25 (29.1)	29 (32.2)	8 (32.0)	23 (18.7)	
Cold and fever	20 (27.4)	13 (15.1)	28 (31.1)	8 (32.0)	44 (35.8)	
Other	11 (15.1)	4 (4.7)	17 (18.9)	5 (20.0)	8 (6.5)	
Dry mouth, n, (%)						<.0001*
Normal	14 (19.2)	37 (43.0)	21 (23.3)	6 (24.0)	46 (37.4)	
Abnormal	49 (67.1)	43 (50.0)	44 (48.9)	13 (52.0)	69 (56.1)	
Other	10 (13.7)	6 (7.0)	25 (27.8)	6 (24.0)	8 (6.5)	
Dyskinesia, n, (%)						<.0001*
Normal	56 (76.7)	59 (68.6)	75 (83.3)	8 (32.0)	97 (78.9)	
Abnormal	11 (15.1)	9 (10.5)	13 (14.4)	5 (20.0)	16 (13.0)	
Other	6 (8.2)	18 (20.9)	2 (2.2)	12 (48.0)	10 (8.1)	
Feces, n, (%)						.0020*
Normal	17 (23.3)	19 (22.1)	32 (35.6)	4 (16.0)	29 (23.6)	
Abnormal	54 (74.0)	66 (76.7)	58 (64.4)	17 (68.0)	89 (72.4)	
Other	2 (2.7)	1 (1.2)	0 (0.0)	4 (16.0)	5 (4.1)	
Gait disturbance, n, (%)						<.0001*
Normal	8 (11.0)	8 (9.3)	4 (4.4)	0 (0.0)	15 (12.2)	
Abnormal	63 (86.3)	75 (87.2)	84 (93.3)	20 (80.0)	108 (87.8)	
Other	2 (2.7)	3 (3.5)	2 (2.2)	5 (20.0)	0 (0.0)	
Postural instability, n, (%)						<.0001*
Normal	16 (21.9)	21 (24.4)	5 (5.6)	1 (4.0)	26 (21.1)	
Abnormal	55 (75.3)	63 (73.3)	76 (84.4)	17 (68.0)	94 (76.4)	
Other	2 (2.7)	2 (2.3)	9 (10.0)	7 (28.0)	3 (2.4)	
Pulse depth, n, (%)						.0061*
Floating	8 (11.0)	12 (14.0)	11 (12.2)	2 (8.0)	11 (8.9)	
Sunken	27 (37.0)	25 (29.1)	27 (30.0)	1 (4.0)	21 (17.1)	
Other	38 (52.1)	49 (57.0)	52 (57.8)	22 (88.0)	91 (74.0)	
Pulse intensity, n, (%)						.0280*
Feeble pulse	4 (5.5)	3 (3.5)	8 (8.9)	0 (0.0)	2 (1.6)	
Solid pulse	15 (20.5)	8 (9.3)	17 (18.9)	2 (8.0)	10 (8.1)	
Feeble and solid pulse	0 (0.0)	1 (1.2)	1 (1.1)	0 (0.0)	0 (0.0)	
None	54 (74.0)	74 (86.0)	64 (71.1)	23 (92.0)	111 (90.2)	
Respiration, n, (%)						<.0001*
Normal	44 (60.3)	39 (45.3)	83 (92.2)	8 (32.0)	25 (20.3)	
Abnormal	1 (1.4)	0 (0.0)	0 (0.0)	0 (0.0)	2 (1.6)	
Other	28 (38.4)	47 (54.7)	7 (7.8)	17 (68.0)	96 (78.0)	
Rigidity, n, (%)						<.0001*
Normal	30 (41.1)	27 (31.4)	30 (33.3)	4 (16.0)	23 (18.7)	
Abnormal	41 (56.2)	55 (64.0)	48 (53.3)	14 (56.0)	94 (76.4)	
Other	2 (2.7)	4 (4.7)	12 (13.3)	7 (28.0)	6 (4.9)	
Sweating, n, (%)						<.0001*
No sweating	7 (9.6)	4 (4.7)	3 (3.3)	0 (0.0)	0 (0.0)	
Unintentional sweating	41 (56.2)	68 (79.1)	49 (54.4)	9 (36.0)	101 (82.1)	
Night sweating	8 (11.0)	13 (15.1)	3 (3.3)	1 (4.0)	19 (15.4)	
Unintentional sweating and Night sweating	8 (11.0)	1 (1.2)	25 (27.8)	7 (28.0)	3 (2.4)	
Other	9 (12.3)	0 (0.0)	10 (11.1)	8 (32.0)	0 (0.0)	
Thirsty, n, (%)						0.0083*
Normal	24 (32.9)	46 (53.5)	25 (27.8)	9 (36.0)	53 (43.1)	
Abnormal	37 (50.7)	33 (38.4)	52 (57.8)	10 (40.0)	61 (49.6)	
Other	12 (16.4)	7 (8.1)	13 (14.4)	6 (24.0)	9 (7.3)	
Tongue coating color, n, (%)						<.0001*
White tongue coating	0 (0.0)	68 (79.1)	67 (74.4)	0 (0.0)	25 (20.3)	
Yellow tongue coating	0 (0.0)	12 (14.0)	16 (17.8)	0 (0.0)	11 (8.9)	
White and yellow tongue coating	0 (0.0)	3 (3.5)	2 (2.2)	0 (0.0)	2 (1.6)	
Other	73 (100.0)	3 (3.5)	5 (5.6)	25 (100.0)	85 (69.1)	
Tongue coating volume, n, (%)						<.0001*
Lack of tongue coating	64 (87.7)	0 (0.0)	0 (0.0)	0 (0.0)	10 (8.1)	
Greasy tongue coating	9 (12.3)	9 (10.5)	21 (23.3)	1 (4.0)	9 (7.3)	
Dry tongue coating	0 (0.0)	4 (4.7)	2 (2.2)	0 (0.0)	2 (1.6)	
Other	0 (0.0)	73 (84.9)	67 (74.4)	24 (96.0)	102 (82.9)	
Tongue color, n, (%)						<.0001*
Light red tongue	25 (34.2)	73 (84.9)	55 (61.1)	0 (0.0)	0 (0.0)	
Pale tongue	0 (0.0)	4 (4.7)	0 (0.0)	0 (0.0)	0 (0.0)	
Red tongue	43 (58.9)	9 (10.5)	34 (37.8)	0 (0.0)	24 (19.5)	
Dark red tongue	4 (5.5)	0 (0.0)	1 (1.1)	0 (0.0)	6 (4.9)	
Other	1 (1.4)	0 (0.0)	0 (0.0)	25 (100.0)	93 (75.6)	
Tongue size, n, (%)						.0002*
Large tongue	0 (0.0)	11 (12.8)	4 (4.4)	0 (0.0)	1 (0.8)	
Other	73 (100.0)	75 (87.2)	86 (95.6)	25 (100.0)	122 (99.2)	
Tremor, n, (%)						.0038*
Normal	18 (24.7)	17 (19.8)	26 (28.9)	3 (12.0)	21 (17.1)	
Abnormal	55 (75.3)	69 (80.2)	64 (71.1)	19 (76.0)	102 (82.9)	
Other	0 (0.0)	0 (0.0)	0 (0.0)	3 (12.0)	0 (0.0)	
Urine, n, (%)						.0005*
Normal	0 (0.0)	0 (0.0)	0 (0.0)	0 (0.0)	0 (0.0)	
Abnormal	60 (82.2)	76 (88.4)	78 (86.7)	13 (52.0)	94 (76.4)	
Other	13 (17.8)	10 (11.6)	12 (13.3)	12 (48.0)	29 (23.6)	
Weakness, n, (%)						<.0001*
Normal	17 (23.3)	23 (26.7)	7 (7.8)	3 (12.0)	42 (34.1)	
Abnormal	42 (57.5)	46 (53.5)	47 (52.2)	8 (32.0)	62 (50.4)	
Other	14 (19.2)	17 (19.8)	36 (40.0)	14 (56.0)	19 (15.4)	

Values are presented as frequency (%) of the corresponding clinical symptom for each EMR-subtype.

EMR subtypes 0 to 4 represent 5 symptom-based clusters identified through hierarchical clustering analysis of PD-related and TEAM symptoms/signs.

EMR = electronic medical record, n = number, PD = Parkinson’s disease.

* *P* < .05 compared with EMR-subtypes.

EMR subtype 0: dry mouth, abnormal feces, sunken pulse, solid pulse, no sweating, lack of tongue coating, red tongue, dark red tongue, and weakness.
EMR subtype 1: cold, floating pulse, feeble and solid pulse, white tongue coating, white and yellow tongue coating, dry tongue coating, light-red tongue, pale tongue, large tongue, and abnormal urine.
EMR subtype 2: abnormal appetite, bitter taste in the mouth, fever, PIGD, feeble pulse, thirst, yellow tongue coating, and greasy tongue coating.
EMR subtype 3: dyskinesia, unintentional sweating, and night sweating.
EMR subtype 4: bradykinesia, cold and fever, abnormal respiration, rigidity, unintentional sweating, night sweating, and tremors.

EMR subtype 1 had the shortest progressive duration and mildest severity, while the other 4 EMR subtypes were moderate stage or longer progressive duration (Table 5).

**Table 5:** Characteristics of patients assigned to each cluster.

	EMR Subtype 0 (n = 73)	EMR Subtype 1 (n = 86)	EMR Subtype 2 (n = 90)	EMR Subtype 3 (n = 25)	EMR Subtype 4 (n = 123)	*P*-value*
Age, mean, SD, yr	70.2 (9.4)	66.2 (9.5)	65.8 (9.8)	67.0 (11.2)	66.1 (8.6)	.0247*
Men, n (%)	16 (21.9)	34 (39.5)	14 (15.6)	8 (32.0)	49 (39.8)	.0003*
Women, n (%)	57 (78.1)	52 (60.5)	76 (84.4)	17 (68.0)	74 (60.2)	
Onset age, mean, SD, y	65.9 (10.3)	62.7 (9.8)	61.8 (10.7)	62.9 (11.4)	61.1 (8.9)	.0248*
UPDRS, mean, SD						
UPDRS II	13.3 (9.3)	10.9 (7.2)	12.9 (7.4)	14.4 (6.6)	12.3 (8.1)	.2776
UPDRS III	18.7 (14.3)	15.5 (9.5)	20.5 (13.1)	21.4 (9.5)	20.0 (14.2)	.0908
H&Y, mean, SD	2.0 (1.2)	1.8 (1.0)	2.5 (1.1)	2.1 (1.1)	2.3 (2.7)	.1287
Progressive duration	4.3 (3.8)	3.4 (3.1)	4.0 (3.7)	3.3 (2.5)	4.8 (4.3)	.0613

EMR = electronic medical record, H&Y = Hoehn and Yahr scale, n = number, NR = not reported, SD = standard deviation, UPDRS = Unified Parkinson’s Disease Rating scale.

Values are presented as mean (standard deviation) or number (%) for each EMR-subtype.

* *P* < .05 compared with EMR-subtypes.

### 3.4. Herbal medicine distribution according to PD progressive duration and symptom-based classification

The therapeutic characteristics of predominantly used herbal medicines for PD were investigated according to PD progressive duration or each cluster. The most frequently used herbal medicine ingredients were Qi-tonifying medicinal, blood-tonifying medicinal, wind-cold dispersing medicinal, and heat-clearing medicinal in the progression (subdivisions A, B, and C) and cluster (EMR subtypes) classifications. However, there was no statistical difference in each group (Tables S4 and S5, Supplemental Digital Content 5).

## 4. Discussion

In this retrospective EMR study of patients with PD, real-world data on PD-associated symptoms, TEAM symptoms/signs, disease duration, and disease severity were analyzed. To the best of our knowledge, this is among the first studies to investigate symptom patterns according to disease severity, progression duration, and TEAM-based clustering using real-world EMR data and a machine-learning approach. Distinct symptom patterns were observed among patients with moderate severity according to disease duration. Additionally, the 4 EMR subtypes of moderate severity, except for EMR subtype 1, demonstrated more frequent symptoms including weakness, sunken pulse, bitter taste, pain, yellow tongue coating, and dyskinesia. Our results suggest that several PD-associated and TEAM systemic symptoms/signs contribute to the characterization of symptom-based PD subtypes. These findings suggest that PD-associated and TEAM systemic symptoms/signs may contribute to the characterization of symptom-based PD subtypes and may reflect clinical heterogeneity related to disease duration and severity. Although further validation is required, these findings may provide preliminary evidence supporting the development of personalized traditional medicine approaches that consider disease severity and progression. The clustering analysis was conducted as a prespecified component of the study design. However, the interpretation of subtype-specific symptom patterns should be considered exploratory and requires validation in independent cohorts.

### 4.1. Key symptoms, considering PD severity and progression over time

The manifestation of clinical symptoms of PD varies depending on the disease progression over time, from the prodromal phase to the early, middle, and late periods.^[37]^ PD-associated disability progressively worsens as it is driven by the exacerbation of motor and non-motor symptoms.^[38]^ Previous reports have shown that dyskinesia generally emerges after approximately 6.5 years of disease progression and chronic levodopa therapy.^[39]^ Similarly, when analyzing the data according to PD severity and progression in this study, subdivision C patients demonstrated dyskinesia compared with patients with a mild stage in subdivision A. These symptoms were observed more frequently in patients with moderate disease severity and longer disease duration. However, because this study was based on retrospective cross-sectional data, no conclusions regarding disease progression or diagnostic utility can be drawn. Future longitudinal studies are required to determine whether these symptom patterns are associated with disease trajectories.

### 4.2. Comprehensive analysis of the newly identified EMR subtypes

Five EMR subtypes were classified using the HCA method regardless of PD progression and severity. EMR subtype 1 had the mildest severity with the shortest progressive duration, while other subtypes exhibited moderate severity compared with EMR subtype 1. This was associated with a high frequency of prominent symptoms, including cold, floating pulse, feeble and solid pulse, white tongue coating, white and yellow tongue coating, dry tongue coating, light-red tongue, pale tongue, large tongue, and abnormal urine in EMR subtype 1. Moreover, some of these symptoms were also observed in mild-stage patients in subdivision A. Although not statistically significant, some symptoms, including cold, floating pulse, solid pulse, dry tongue coating, light-red tongue, pale tongue, and large tongue, were more commonly observed in patients with a mild stage in subdivision A rather than those with a mild stage in subdivision C (Table S3, Supplemental Digital Content 3). Therefore, these findings suggest that certain systemic symptoms/signs may be shared across the newly identified TEAM subtypes despite differences in disease duration and severity. In contrast with subtype 1, the other EMR subtypes were classified as having a moderate stage or a long progressive duration in subdivision C. EMR subtype 4, which had the longest duration of progression but not the highest disease severity, demonstrated a high frequency of prominent symptoms, including tremor, rigidity, bradykinesia, cold and fever, unintentional sweating, night sweating, and abnormal respiration. However, EMR subtype 2, which had the highest disease severity based on the H&Y scale but not the longest duration of progression, exhibited a high frequency of prominent symptoms, including PIGD, fever, thirst, bitter taste, abnormal appetite, yellow tongue coating, greasy tongue coating, and feeble pulse. Previously, PD was classified into 2 subtypes based on tremors or PIGD.^[40]^ Previous studies have reported that PIGD-dominant PD is generally associated with more severe clinical outcomes than tremor-dominant PD.^[41]^ In the present study, EMR subtype 2 showed a higher frequency of PIGD-related symptoms, whereas EMR subtype 4 demonstrated a predominance of tremor-related symptoms. Although these observations are broadly consistent with previous subtype classifications, the cross-sectional design of the current study does not permit conclusions regarding prognosis or disease progression. However, each symptom was judged based on frequency rather than the severity score. Moreover, this study had a small sample size; thus, further studies are needed to verify this.

Our study had the following limitations: First, as this study was a retrospective review of real-world data, quality control was limited because the raw data could not be strictly controlled, and a substantial amount of missing data was present; furthermore, there was much missing data. In addition, the analysis was performed by symptom frequency rather than by the severity evaluation score for each symptom. In the future, we will use well-designed observational study methods to assess the SD of PD. Second, the retrospective data used were not based on the longitudinal sequential progression for each patient; instead, the retrospective data were compared with the cross-sectional data of patients with a specific disease progression duration. Third, we intended to analyze the herbal medicine ingredients to assess the subtypes; however, these details could not be extracted from the records because of restrictions on prescription information. More studies involving herbal medicine prescription by subtype are required to personalize therapy in the future. Fourth, in this study, several potential confounding factors, including medication use and treatment interventions, were not consistently available across all records and, therefore, could not be fully controlled. In addition, age, sex, and other clinical factors may have influenced the observed symptom patterns. Future studies using standardized clinical datasets and multivariable analytical approaches are needed to validate these findings. Fifth, correction for multiple comparisons was not applied because the analyses were exploratory. Therefore, the possibility of false-positive findings cannot be excluded, and the observed associations should be validated in larger independent cohorts using prespecified multiplicity-adjustment procedures. Finally, a low number of patients met the inclusion criteria. Thus, we were unable to observe some symptoms because of the study’s low statistical power. To overcome these limitations, we plan to conduct a prospective observational clinical study based on the present study. Although further validation is required, these findings may provide preliminary evidence supporting the development of personalized traditional medicine approaches that consider disease severity and progression. The clustering analysis was conducted as a prespecified component of the study design. However, the interpretation of subtype-specific symptom patterns should be considered exploratory and hypothesis-generating, rather than validated clinical conclusions, and requires confirmation in independent cohorts and prospective longitudinal studies.

PD has a spectrum of PD-associated symptoms and TEAM symptoms/signs with high-dimensional variability that are unintuitive and difficult to interpret.^[2,42]^ Therefore, methodologies involving an unsupervised learning-based clustering approach with dimension reduction may be useful in developing a PD classification system using symptom data, including TEAM syndromes in PD. Considering PD severity and disease duration, several PD-associated and TEAM symptoms/signs demonstrated distinct distribution patterns across patient subgroups. However, because this study was based on retrospective cross-sectional EMR data, these findings should be considered exploratory and hypothesis-generating rather than prognostic indicators. Future prospective studies are needed to determine whether these symptom patterns may have implications for individualized traditional medicine approaches tailored to disease severity and progression. These preliminary findings provide a basis for future hypothesis generation and warrant validation in independent cohorts and longitudinal studies. Ultimately, these findings may contribute to the refinement of TEAM diagnostic criteria for patients with PD.

## Author contributions

**Conceptualization:** Jiyun Cha, Seong-Uk Park, Jung-Hee Jang.

**Formal analysis:** HuiYan Zhao, Ojin Kwon.

**Methodology:** HuiYan Zhao, Ojin Kwon, Jae Young Jang, Gyu Ri Jeon, Ye-Chae Hwang, Eunbyul Cho, Jiyun Cha, Eun Kyoung Ahn, Jung-Hee Jang.

**Supervision:** Seong-Uk Park, Jung-Hee Jang.

**Writing – original draft:** HuiYan Zhao, Jung-Hee Jang.

**Writing – review & editing:** Jung-Hee Jang.












