# Supplementary material for: Symptom-based classification of Parkinson’s disease using retrospective electronic medical records: A STROBE-compliant cross-sectional study
Source: Medicine (Baltimore). 2026 Sep 18;105(38):e50608. doi: 10.1097/MD.0000000000050608 (PMC13593295; doi:10.1097/MD.0000000000050608)

Supplemental Figure 1. Radar plots for variables of the five subtypes of Parkinson's disease.

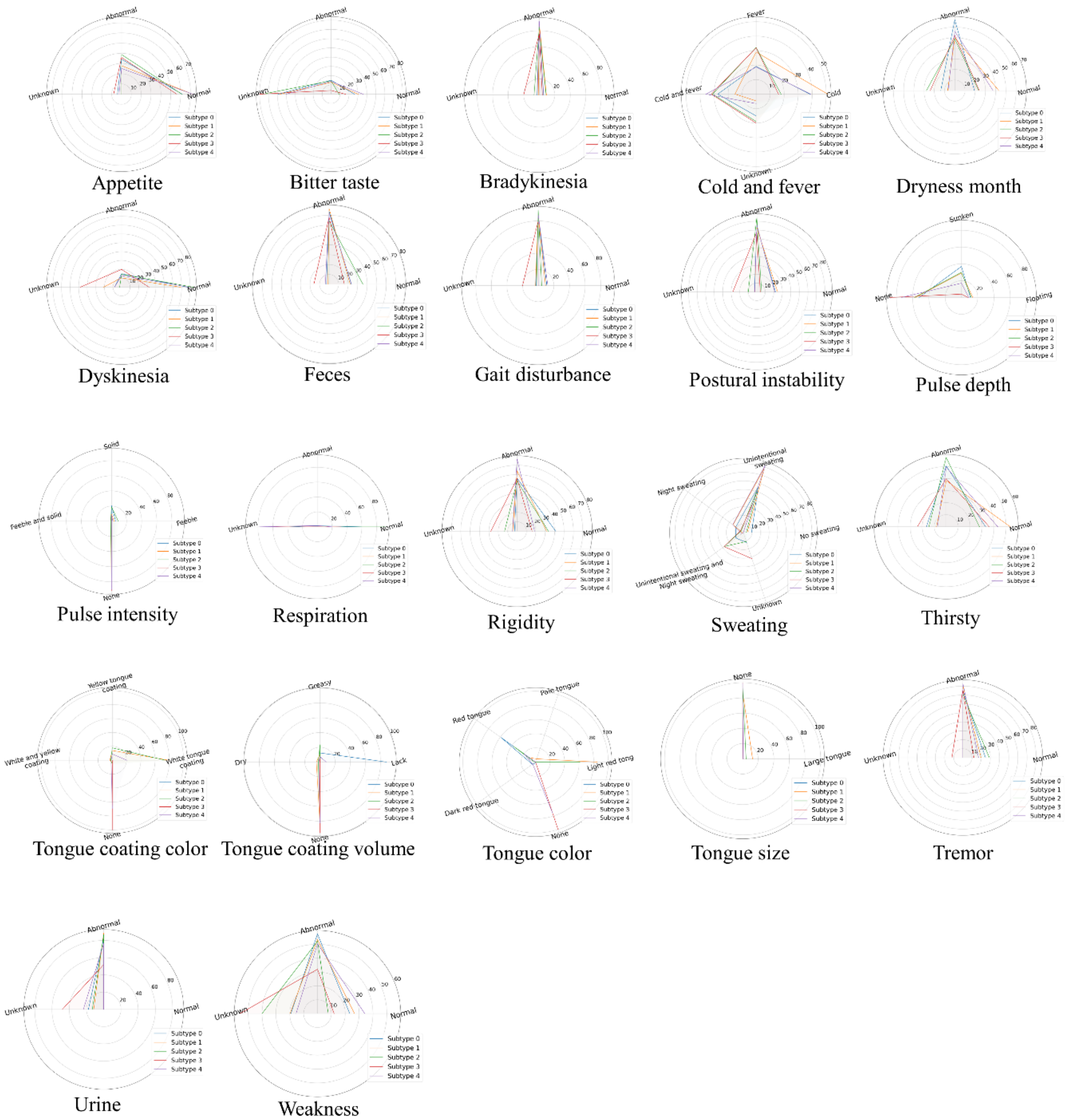

Supplement: Supplementary file 1 [file medi-105-e50608-s004.pdf]
