# Supplementary material for: Symptom-based classification of Parkinson’s disease using retrospective electronic medical records: A STROBE-compliant cross-sectional study
Source: Medicine (Baltimore). 2026 Sep 18;105(38):e50608. doi: 10.1097/MD.0000000000050608 (PMC13593295; doi:10.1097/MD.0000000000050608)
Supplement: Supplementary file 2 [file medi-105-e50608-s001.docx]

Supplemental Table 1. Variables and their corresponding codes

| Principal feature | Variable |  | Content | Code |
| --- | --- | --- | --- | --- |
| Appetite | Normal |  | Normal | 1 |
|  | Increased appetite; loss of appetite; no appetite; no ingestion of food |  | Abnormal | 2 |
| Bradykinesia | Normal |  | Normal | 1 |
|  | Abnormal |  | Abnormal | 2 |
| Bitter taste in mouth | Normal |  | Normal | 1 |
|  | Abnormal |  | Abnormal | 2 |
| Coughing | Normal |  | Normal | 1 |
|  | Abnormal |  | Abnormal | 2 |
| Cold and fever | Avoidance of cold temperatures; avoidance of cold temperatures and fear of windiness |  | Cold | 1 |
|  | Avoidance of warm temperatures; avoidance of warm temperatures and fear of windiness |  | Fever | 2 |
|  | Avoidance of cold temperatures; avoidance of warm temperatures and fear of windiness |  | Cold and fever | 3 |
| Dyskinesia | Normal |  | Normal | 1 |
|  | Abnormal |  | Abnormal | 2 |
| Dry mouth | Normal |  | Normal | 1 |
|  | Abnormal |  | Abnormal | 2 |
| Dizziness | Normal |  | Normal | 1 |
|  | Abnormal |  | Abnormal | 2 |
| Digestion | Normal |  | Normal | 1 |
|  | Nausea; vomiting; longevity; stomachache; feeling full and indigestion; pain after eating |  | Abnormal | 2 |
| Feces | Normal |  | Normal | 1 |
|  | Smooth; hardened; rectal pressure; hardened and rectal pressure; blood; hardened and smooth; smooth and rectal pressure; hardened and blood; hardened, blood, rectal pressure; constipation |  | Abnormal | 2 |
| Finger counting | No |  | Normal | 1 |
|  | Yes |  | Abnormal | 2 |
| Fullness in the chest | Normal |  | Normal | 1 |
|  | Abnormal |  | Abnormal | 2 |
| Gait disturbance | No |  | Normal | 1 |
|  | Yes |  | Abnormal | 2 |
| Headache | Normal |  | Normal | 1 |
|  | Abnormal |  | Abnormal | 2 |
| Postural instability | No; able to stand without stooped posture; able to stand |  | Normal | 1 |
|  | Yes; able to stand with stooped posture; unable to stand with stooped posture; unable to stand; unable to stand with stooped posture |  | Abnormal | 2 |
| Phlegm | No |  | Normal | 1 |
|  | Yes; white; yellow; white and yellow; throat discomfort; white; yellow; and throat discomfort |  | Abnormal | 2 |
| Pain | Normal |  | Normal | 1 |
|  | Abnormal |  | Abnormal | 2 |
| Pulse depth | Floating pulse; hollow pulse |  | Floating pulse | 1 |
|  | Sunken pulse; Weak pulse |  | Sunken pulse | 2 |
| Pulse rate | Slow pulse; moderate pulse; rough pulse |  | Slow pulse; | 1 |
|  | Rapid pulse |  | Rapid pulse | 2 |
| Pulse intensity | Feeble pulse; fine pulse; intermittent pulse |  | Feeble pulse | 1 |
|  | Wiry pulse; tight pulse; slippery pulse |  | Solid pulse | 2 |
| Respiration | Normal |  | Normal | 1 |
|  | Abnormal |  | Abnormal | 2 |
| Rigidity | Normal |  | Normal | 1 |
|  | Abnormal |  | Abnormal | 2 |
| Speech disorder | Normal |  | Normal | 1 |
|  | Abnormal |  | Abnormal | 2 |
| Sleep disorder | Normal |  | Normal | 1 |
|  | Difficulty falling (back) asleep; mid-sleep awakenings or arousal; excessive dreaming; (mid-sleep awakenings or arousal) and (difficulty falling back asleep); excessive dreaming and difficulty falling back asleep; mid-sleep awakenings or arousal and excessive dreaming; mid-sleep awakenings or arousal and excessive dreaming) |  | Abnormal | 2 |
| Swallowing | Yes |  | Abnormal | 2 |
| Sensory/paresthesia | No |  | Normal | 1 |
|  | Yes |  | Abnormal | 2 |
| Sweating | No |  | No sweating | 1 |
|  | Yes; unintentional sweating |  | Unintentional sweating | 2 |
|  | Night sweating; night sweating and yes |  | Night sweating | 3 |
|  | Unintentional sweating and night sweating |  | Unintentional sweating and night sweating | 4 |
| Tremor | Normal |  | Normal | 1 |
|  | Abnormal |  | Abnormal | 2 |
| Tongue movement | No |  | Normal | 1 |
|  | Yes |  | Abnormal | 2 |
| Tongue color | Light-red tongue |  | Light-red tongue | 1 |
|  | Pale tongue |  | Pale tongue | 2 |
|  | Red tongue |  | Red tongue | 3 |
|  | Dark red tongue |  | Dark red tongue | 4 |
|  | None |  | None | 5 |
| Tongue size | Large |  | Large tongue | 1 |
|  | None |  | None | 2 |
| Tongue coating color | White tongue coating |  | White | 1 |
|  | Yellow tongue coating |  | Yellow | 2 |
|  | White and yellow coating |  | White and yellow | 3 |
|  | None |  | None | 4 |
| Tongue coating volume | Lack of tongue coating |  | Lacking | 1 |
|  | Greasy tongue coating |  | Greasy | 2 |
|  | Dry tongue coating |  | Dry | 3 |
| Thirsty | Normal |  | Normal | 1 |
|  | Abnormal |  | Abnormal | 2 |
| Urine | Normal |  | Normal | 1 |
|  | Symptoms of urinary incontinence; symptoms of urinary incontinence and urinary hesitancy |  | Abnormal | 2 |
| Weakness | No |  | Normal | 1 |
|  | Yes |  | Abnormal | 2 |
