# Supplementary material for: Symptom-based classification of Parkinson’s disease using retrospective electronic medical records: A STROBE-compliant cross-sectional study
Source: Medicine (Baltimore). 2026 Sep 18;105(38):e50608. doi: 10.1097/MD.0000000000050608 (PMC13593295; doi:10.1097/MD.0000000000050608)
Supplement: Supplementary file 3 [file medi-105-e50608-s002.docx]

Supplemental Table 2. Demographic characteristics and disease severity according to prevalence and progressive duration

|  | Within 3 years | 3 to 7 years | Over 7 years | P-value |
| --- | --- | --- | --- | --- |
| Total (n=397) | (n=219) | (n=95) | (n=83) |  |
| Men, n (%) | 81 (37.0) | 16 (16.8) | 24 (28.9) | 0.0013* |
| Women, n (%) | 138 (63.0) | 79 (83.2) | 59 (71.1) |  |
| Age (years), mean, SD | 66.9 (9.9) | 66.5 (8.7) | 67.2 (9.4) | 0.9034 |
| Age at onset (years), mean, SD | 65.4 (9.8) | 61.4 (8.7) | 56.8 (9.3) | <.0001* |
| Progressive duration (years) mean, SD | 1.5 (1.0) | 5.0 (0.8) | 10.1 (2.9) | <.0001* |
| H&Y, mean, SD | 1.8 (1.1) | 2.2 (1.2) | 2.3 (1.2) | 0.0190* |
| UPDRS Ⅱ, mean, SD | 11.4 (7.6) | 13.0 (7.6) | 14.5 (8.9) | 0.0137* |
| UPDRS Ⅲ, mean, SD | 17.6 (11.5) | 20.3 (14.3) | 21.0 (14.5) | 0.0987 |
| Mild (n=177) | (n=113) | (n=32) | (n=32) |  |
| Men, n (%) | 46, (40.7) | 10, (31.3) | 12, (37.5) | 0.6434 |
| Women, n (%) | 67, (59.3) | 22, (68.8) | 20, (62.5) |  |
| Age (years), mean, SD | 64.6 (9.9) | 64.6 (7.1) | 65.8 (8.1) | 0.8133 |
| Age at onset (years), mean, SD | 63.0 (9.8) | 59.6 (7.4) | 54.7 (8.6) | <.0001* |
| Progressive duration (years), mean, SD | 1.5 (1.0) | 5.0 (0.7) | 10.3 (2.6) | <.0001* |
| H&Y, mean, SD | 1.3 (0.5) | 1.5 (0.5) | 1.4 (0.6) | 0.0989 |
| UPDRS Ⅱ, mean, SD | 8.1 (5.2) | 9.5 (4.3) | 10.6 (5.8) | 0.0468* |
| UPDRS Ⅲ, mean, SD | 13.1 (7.7) | 15.6 (9.0) | 13.7 (9.7) | 0.3385 |
| Moderate (n=78) | (n=38) | (n=19) | (n=21) |  |
| Men, n (%) | 16, (42.1) | 3, (15.8) | 6, (28.6) | 0.1377 |
| Women, n (%) | 22, (57.9) | 16, (84.2) | 15, (71.4) |  |
| Age (years), mean, SD | 70.1 (8.7) | 70.3 (8.6) | 70.3 (9.9) | 0.9942 |
| Age at onset (years), mean, SD | 68.6 (8.6) | 64.6 (8.4) | 60.9 (9.8) | 0.0075* |
| Progressive duration (years), mean, SD | 1.6 (1.1) | 4.7 (0.8) | 9.5 (2.2) | <.0001* |
| H&Y, mean, SD | 3.5 (0.7) | 3.5 (0.8) | 3.5 (0.7) | 0.9524 |
| UPDRS Ⅱ, mean, SD | 18.7 (7.9) | 18.5 (10.0) | 19.9 (8.8) | 0.8596 |
| UPDRS Ⅲ, mean, SD | 28.9 (12.9) | 30.3 (19.0) | 33.8 (13.6) | 0.4846 |

H&Y: Hoehn and Yahr scale; n: number; SD: standard deviation; UPDRS: Unified Parkinson’s Disease Rating scale
