# Supplementary material for: Symptom-based classification of Parkinson’s disease using retrospective electronic medical records: A STROBE-compliant cross-sectional study
Source: Medicine (Baltimore). 2026 Sep 18;105(38):e50608. doi: 10.1097/MD.0000000000050608 (PMC13593295; doi:10.1097/MD.0000000000050608)
Supplement: Supplementary file 4 [file medi-105-e50608-s003.docx]

Supplemental Table 3. Frequencies of all symptoms or signs according to progressive duration and disease severity

| Parameter | Estimate | Standard | Wald | P-value |
| --- | --- | --- | --- | --- |
|  |  | Error | Chi-square |  |
| Subdivision A (mild) vs. subdivision C (mild) | | | | |
| Bitter taste | 1.8638 | 1.059 | 3.0974 | 0.0784 |
| Bradykinesia | -10.3822 | 241.3 | 0.0019 | 0.9657 |
| Cold | 0.3972 | 0.6738 | 0.3474 | 0.5556 |
| Cold and fever | -0.1108 | 0.6701 | 0.0273 | 0.8687 |
| Dark red tongue | -0.6131 | 1.264 | 0.2353 | 0.6276 |
| Digestion | 11.165 | 174.9 | 0.0041 | 0.9491 |
| Dry tongue coating volume | 12.242 | 463.8 | 0.0007 | 0.9789 |
| Dry mouth | -0.5265 | 1.124 | 0.2194 | 0.6395 |
| Dyskinesia | -3.2386 | 0.9445 | 11.7568 | 0.0006* |
| Feces | 0.393 | 0.8942 | 0.1932 | 0.6603 |
| Feeble pulse | -1.1866 | 1.43 | 0.6885 | 0.4067 |
| Fever | 0.821 | 0.7943 | 1.0683 | 0.3013 |
| Floating pulse | 12.2437 | 327.9 | 0.0014 | 0.9702 |
| Gait disturbance | 0.4884 | 1.2443 | 0.154 | 0.6947 |
| Greasy tongue coating volume | -0.1238 | 0.6973 | 0.0315 | 0.8591 |
| Lack tongue coating volume | -0.9123 | 0.4897 | 3.4703 | 0.0625 |
| Large tongue | 0.1335 | 0.8172 | 0.0267 | 0.8702 |
| Light-red tongue | 0.1047 | 0.4713 | 0.0494 | 0.8241 |
| Night sweating | 0.1823 | 1.169 | 0.0243 | 0.8761 |
| No sweating | 13.6651 | 655.9 | 0.0004 | 0.9834 |
| Non-appetite | -0.0528 | 0.4443 | 0.0141 | 0.9055 |
| Non-bitter taste | 0.471 | 0.4474 | 1.1083 | 0.2924 |
| Non-bradykinesia | -10.7488 | 241.3 | 0.002 | 0.9645 |
| Non-digestion | 11.8572 | 174.9 | 0.0046 | 0.946 |
| Non-dry mouth | -0.0831 | 1.1495 | 0.0052 | 0.9423 |
| Non-dyskinesia | 0.3238 | 0.618 | 0.2745 | 0.6003 |
| Non-feces | 1.5753 | 1.0572 | 2.2201 | 0.1362 |
| Non-gait disturbance | 1.0561 | 1.3392 | 0.6218 | 0.4304 |
| Non-pain | -0.4139 | 1.1947 | 0.12 | 0.729 |
| Non-postural instability | 0.8708 | 1.2784 | 0.464 | 0.4958 |
| Non-respiration | 0.0096 | 0.4452 | 0.0005 | 0.9829 |
| Non-rigidity | -0.6131 | 1.1712 | 0.274 | 0.6006 |
| Non-sleep disorder | -9.0556 | 207 | 0.0019 | 0.9651 |
| Non-thirsty | 0.8507 | 0.6321 | 1.8115 | 0.1783 |
| Non-tremor | -0.4818 | 0.4594 | 1.1002 | 0.2942 |
| Non-weakness | -0.2283 | 0.541 | 0.1781 | 0.673 |
| Pain | -0.4711 | 0.4111 | 1.3129 | 0.2519 |
| Pale tongue | 12.3588 | 655.9 | 0.0004 | 0.985 |
| Postural instability | 0.4353 | 1.2491 | 0.1215 | 0.7275 |
| Rapid pulse | 1.1898 | 1.0654 | 1.2471 | 0.2641 |
| Red tongue | -0.39 | 0.5223 | 0.5575 | 0.4553 |
| Rigidity | 0.0881 | 1.1466 | 0.0059 | 0.9387 |
| Sleep disorder | -9.495 | 207 | 0.0021 | 0.9634 |
| Slow pulse | -0.2911 | 0.863 | 0.1138 | 0.7359 |
| Solid pulse | 0.9534 | 0.777 | 1.5054 | 0.2198 |

| Speech disorder | 0.0034 | 0.4077 | 0.0001 | 0.9933 |
| --- | --- | --- | --- | --- |
| Sunken pulse | -0.9289 | 0.4534 | 4.1973 | 0.0405* |
| Swallowing | -0.2495 | 0.4172 | 0.3575 | 0.5499 |
| Thirsty | 0.6539 | 0.6405 | 1.0423 | 0.3073 |
| Unintentional sweating | 1.5404 | 1.0285 | 2.2433 | 0.1342 |
| Unintentional sweating and night sweating | 0.2231 | 1.2042 | 0.0343 | 0.853 |
| Urine | -0.2079 | 0.5064 | 0.1684 | 0.6815 |
| Weakness | 0.3507 | 0.5362 | 0.4278 | 0.5131 |
| White and yellow tongue coating color | 12.5337 | 535.5 | 0.0005 | 0.9813 |
| White tongue coating color | 0.2796 | 0.4365 | 0.4103 | 0.5218 |
| Yellow tongue coating color | 0.1214 | 0.842 | 0.0208 | 0.8854 |
| Subdivision A (mild) vs. subdivision C (moderate) | | | | |
| Appetite | 13.8226 | 247.1 | 0.0031 | 0.9554 |
| Bitter taste | -0.0233 | 0.7137 | 0.0011 | 0.974 |
| Bradykinesia | -10.337 | 279.1 | 0.0014 | 0.9705 |
| Cold | -0.2006 | 1.1459 | 0.0307 | 0.861 |
| Cold and fever | -1.2347 | 1.1055 | 1.2474 | 0.264 |
| Dark red tongue | -1.0986 | 1.2829 | 0.7334 | 0.3918 |
| Digestion | 13.7273 | 349.4 | 0.0015 | 0.9687 |
| Dry tongue volume | -0.4676 | 1.1571 | 0.1633 | 0.6861 |
| Dry mouth | -0.3155 | 1.1291 | 0.0781 | 0.7799 |
| Dyskinesia | -3.3928 | 1.0962 | 9.5793 | 0.002* |
| Feces | -10.2825 | 197.4 | 0.0027 | 0.9585 |
| Feeble pulse | 12.2929 | 1073 | 0.0001 | 0.9909 |
| Fever | -0.7884 | 1.1536 | 0.4671 | 0.4943 |
| Floating pulse | -1.6933 | 0.6607 | 6.5691 | 0.0104* |
| Gait disturbance | -10.5236 | 279.1 | 0.0014 | 0.9699 |
| Greasy tongue volume | -0.5546 | 0.7164 | 0.5993 | 0.4388 |
| Lack tongue volume | -0.5321 | 0.6369 | 0.6981 | 0.4034 |
| Large tongue | 12.347 | 379.4 | 0.0011 | 0.974 |
| Light-red tongue | 0.5355 | 0.6482 | 0.6824 | 0.4088 |
| Night sweating | -0.6931 | 1.354 | 0.2621 | 0.6087 |
| No sweating | 13.2633 | 758.7 | 0.0003 | 0.9861 |
| Non-appetite | 13.3864 | 247.1 | 0.0029 | 0.9568 |
| Non-bitter taste | -0.1997 | 0.5182 | 0.1485 | 0.6999 |
| Non-bradykinesia | -9.6538 | 279.1 | 0.0012 | 0.9724 |
| Non-digestion | 13.3587 | 349.4 | 0.0015 | 0.9695 |
| Non-dry mouth | 1.1206 | 1.247 | 0.8075 | 0.3689 |
| Non-dyskinesia | -0.0817 | 0.808 | 0.0102 | 0.9195 |
| Non-feces | -10.3809 | 197.4 | 0.0028 | 0.9581 |
| Non-gait disturbance | 0 | 291 | 0 | 0.9999 |
| Non-pain | -1.1872 | 1.2316 | 0.9291 | 0.3351 |
| Non-postural instability | -7.1952 | 169.3 | 0.0018 | 0.9661 |
| Non-respiration | -0.3097 | 0.5132 | 0.3641 | 0.5462 |
| Non-rigidity | -9.3915 | 197.4 | 0.0023 | 0.962 |
| Non-sleep disorder | 2.1207 | 1.5406 | 1.8949 | 0.1687 |
| Non-thirsty | 0.2708 | 1.1524 | 0.0552 | 0.8142 |
| Non-tremor | 0.0269 | 0.6043 | 0.002 | 0.9644 |
| Non-weakness | -1.2185 | 1.1282 | 1.1665 | 0.2801 |
| Pain | -0.993 | 0.5241 | 3.5902 | 0.0581 |
| Pale tongue | -1.0986 | 1.2829 | 0.7334 | 0.3918 |
| Postural instability | -9.7326 | 169.3 | 0.0033 | 0.9542 |
| Rapid pulse | -0.9241 | 0.6501 | 2.0203 | 0.1552 |
| Red tongue | -0.7419 | 0.582 | 1.6253 | 0.2024 |
| Respiration | -15.5419 | 949.8 | 0.0003 | 0.9869 |
| Rigidity | -10.482 | 197.4 | 0.0028 | 0.9576 |
| Sleep disorder | 1.6331 | 1.4389 | 1.2882 | 0.2564 |
| Slow pulse | -1.4248 | 0.7843 | 3.3007 | 0.0692 |
| Solid pulse | 0.0711 | 0.6769 | 0.011 | 0.9164 |
| Speech disorder | -1.5389 | 0.5469 | 7.9178 | 0.0049* |
| Sunken pulse | -1.0647 | 0.5777 | 3.3969 | 0.0653 |
| Swallowing | -0.665 | 0.4813 | 1.9087 | 0.1671 |
| Thirsty | -1.0986 | 1.0954 | 1.0058 | 0.3159 |
| Unintentional sweating | 1.4069 | 1.2624 | 1.2421 | 0.2651 |
| Unintentional sweating and night sweating | 0.2231 | 1.4832 | 0.0226 | 0.8804 |
| Urine | -0.1884 | 0.6002 | 0.0986 | 0.7535 |
| Weakness | -1.8182 | 1.0621 | 2.9307 | 0.0869 |
| White and yellow tongue coating | 12.1319 | 619.5 | 0.0004 | 0.9844 |
| White tongue coating | 0.0973 | 0.5542 | 0.0308 | 0.8607 |
| Yellow tongue coating | -1.4881 | 0.6775 | 4.8245 | 0.0281* |
| Subdivision A (moderate) vs. subdivision C (moderate) | | | | |
| Appetite | 1.1787 | 1.1519 | 1.047 | 0.3062 |
| Bitter taste | -0.0282 | 0.5933 | 0.0023 | 0.9621 |
| Bradykinesia | -11.7071 | 474 | 0.0006 | 0.9803 |
| Cold | -0.3567 | 1.2335 | 0.0836 | 0.7725 |
| Cold and fever | -1.1239 | 1.1945 | 0.8853 | 0.3468 |
| Dark red tongue | -14.0101 | 804.9 | 0.0003 | 0.9861 |
| Digestion | 0.9162 | 1.533 | 0.3572 | 0.5501 |
| Dry mouth | -1.084 | 1.1629 | 0.8689 | 0.3512 |
| Dyskinesia | -0.2719 | 1.0494 | 0.0672 | 0.7955 |
| Feces | -10.5338 | 203.3 | 0.0027 | 0.9587 |
| Feeble pulse | 12.8115 | 552.6 | 0.0005 | 0.9815 |
| Fever | -1.0498 | 1.2621 | 0.6919 | 0.4055 |
| Floating pulse | -1.5041 | 0.8188 | 3.3746 | 0.0662 |
| Gait disturbance | -11.7833 | 474 | 0.0006 | 0.9802 |
| Large tongue | 12.7559 | 781.4 | 0.0003 | 0.987 |
| Light-red tongue | 0.7577 | 0.71 | 1.1387 | 0.2859 |
| Night sweating | -0.9445 | 1.2817 | 0.543 | 0.4612 |
| No sweating | 12.2237 | 781.4 | 0.0002 | 0.9875 |
| Non-appetite | 0.4274 | 1.0536 | 0.1646 | 0.685 |
| Non-bitter taste | 0.1054 | 0.8097 | 0.0169 | 0.8965 |
| Non-bradykinesia | -24.7038 | 680.4 | 0.0013 | 0.971 |
| Non-digestion | 0.5232 | 1.449 | 0.1304 | 0.718 |
| Non-dry mouth | -0.087 | 1.2939 | 0.0045 | 0.9464 |
| Non-dyskinesia | 0.4055 | 0.9766 | 0.1724 | 0.678 |
| Non-feces | -12.0154 | 203.3 | 0.0035 | 0.9529 |
| Non-gait disturbance | 0 | 670.3 | 0 | 0.9999 |
| Non-pain | -14.6808 | 804.9 | 0.0003 | 0.9854 |
| Non-postural instability | -24.7038 | 680.4 | 0.0013 | 0.971 |
| Non-respiration | 0.3006 | 0.5725 | 0.2757 | 0.5995 |
| Non-rigidity | -9.3764 | 203.3 | 0.0021 | 0.9632 |
| Non-sleep disorder | 0 | 1.633 | 0 | 0.9999 |
| Non-thirsty | -0.7339 | 1.2179 | 0.3631 | 0.5468 |
| Non-tremor | -11.3223 | 287.5 | 0.0016 | 0.9686 |
| Non-weakness | -1.1394 | 1.2349 | 0.8513 | 0.3562 |
| Pain | -1.1658 | 0.5883 | 3.9271 | 0.0475* |
| Pale tongue | -14.0101 | 804.9 | 0.0003 | 0.9861 |
| Postural instability | -11.7071 | 474 | 0.0006 | 0.9803 |
| Rapid pulse | -0.1686 | 0.7062 | 0.057 | 0.8113 |
| Red tongue | -0.6286 | 0.6909 | 0.8277 | 0.3629 |
| Respiration | -13.9076 | 804.9 | 0.0003 | 0.9862 |
| Rigidity | -11.1757 | 203.3 | 0.003 | 0.9562 |
| Sleep disorder | 0.6931 | 1.4451 | 0.2301 | 0.6315 |
| Slow pulse | -1.1332 | 0.9691 | 1.3674 | 0.2423 |
| Solid pulse | 0.1823 | 0.7674 | 0.0564 | 0.8122 |
| Speech disorder | -0.2652 | 0.6249 | 0.1801 | 0.6713 |
| Sunken pulse | -0.7056 | 0.6548 | 1.1612 | 0.2812 |
| Swallowing | 0.2007 | 0.5445 | 0.1358 | 0.7125 |
| Thirsty | -0.7339 | 1.2179 | 0.3631 | 0.5468 |
| Tremor | -10.659 | 287.5 | 0.0014 | 0.9704 |
| Unintentional sweating | -0.4055 | 1.2076 | 0.1127 | 0.7371 |
| Unintentional sweating and night sweating | -0.6931 | 1.472 | 0.2217 | 0.6377 |
| Urine | 0.2271 | 0.7119 | 0.1017 | 0.7498 |
| Weakness | -1.0986 | 1.1431 | 0.9236 | 0.3365 |
| White tongue coating | 0.1278 | 0.6155 | 0.0431 | 0.8355 |
| Yellow tongue coating | -2.3979 | 1.1599 | 4.2736 | 0.0387* |
| Subdivision A (moderate) vs. subdivision C (mild) | | | | |
| Appetite | -10.8464 | 192.6 | 0.0032 | 0.9551 |
| Bitter taste | 1.9919 | 1.1257 | 3.131 | 0.0768 |
| Bradykinesia | -10.9354 | 272.4 | 0.0016 | 0.968 |
| Cold | 0.2412 | 0.8138 | 0.0878 | 0.767 |
| Cold and fever | 0 | 0.8086 | 0 | 0.9999 |
| Dark red tongue | -13.3586 | 740.8 | 0.0003 | 0.9856 |
| Digestion | -0.2624 | 1.4754 | 0.0316 | 0.8589 |
| Dry mouth | -1.2953 | 1.1581 | 1.2511 | 0.2633 |
| Dyskinesia | -0.1178 | 0.8898 | 0.0175 | 0.8947 |
| Feces | 0.2007 | 1.0331 | 0.0377 | 0.846 |
| Feeble pulse | 0.6592 | 1.2521 | 0.2772 | 0.5985 |
| Fever | 0.5596 | 0.9449 | 0.3507 | 0.5537 |
| Floating pulse | 12.9629 | 427.4 | 0.0009 | 0.9758 |
| Gait disturbance | 0.2877 | 1.4369 | 0.0401 | 0.8413 |
| Greasy tongue volume | 0.1743 | 0.7868 | 0.0491 | 0.8246 |
| Lack tongue volume | -0.9242 | 0.6299 | 2.1526 | 0.1423 |
| Large tongue | -0.9028 | 1.2491 | 0.5224 | 0.4698 |
| Light-red tongue | 0.3269 | 0.5533 | 0.3491 | 0.5546 |
| Night sweating | -0.069 | 1.0845 | 0.004 | 0.9493 |
| No sweating | 12.8087 | 740.3 | 0.0003 | 0.9862 |
| Non-appetite | -11.2142 | 192.6 | 0.0034 | 0.9536 |
| Non-bitter taste | 0.6425 | 0.5325 | 1.4559 | 0.2276 |
| Non-bradykinesia | -22.4297 | 304.5 | 0.0054 | 0.9413 |
| Non-digestion | 0.4055 | 1.4466 | 0.0786 | 0.7793 |
| Non-dry mouth | -1.291 | 1.2004 | 1.1566 | 0.2822 |
| Non-dyskinesia | 0.8109 | 0.8264 | 0.963 | 0.3264 |
| Non-feces | 0 | 1.291 | 0 | 0.9999 |
| Non-gait disturbance | -1.3863 | 1.8028 | 0.5913 | 0.4419 |
| Non-pain | -13.7416 | 740.8 | 0.0003 | 0.9852 |
| Non-postural instability | -13.2155 | 246.9 | 0.0029 | 0.9573 |
| Non-respiration | 0.6198 | 0.5124 | 1.4632 | 0.2264 |
| Non-rigidity | -0.5389 | 1.2864 | 0.1755 | 0.6753 |
| Non-sleep disorder | -11.725 | 272.4 | 0.0019 | 0.9657 |
| Non-thirsty | -0.1542 | 0.7448 | 0.0428 | 0.836 |
| Non-tremor | -12.0251 | 272.4 | 0.0019 | 0.9648 |
| Non-weakness | -0.149 | 0.7381 | 0.0408 | 0.84 |
| Pain | -0.6439 | 0.4903 | 1.7243 | 0.1891 |
| Postural instability | 0.5199 | 1.4396 | 0.1304 | 0.718 |
| Rapid pulse | 1.9459 | 1.1008 | 3.1247 | 0.0771 |
| Red tongue | -0.2766 | 0.6414 | 0.186 | 0.6663 |
| Rigidity | -0.5465 | 1.2641 | 0.1869 | 0.6655 |
| Sleep disorder | -10.9836 | 272.4 | 0.0016 | 0.9678 |
| Slow pulse | 0 | 1.0339 | 0 | 1 |
| Solid pulse | 1.0647 | 0.857 | 1.5434 | 0.2141 |
| Speech disorder | 1.2773 | 0.5074 | 6.336 | 0.0118* |
| Sunken pulse | -0.5698 | 0.5482 | 1.0801 | 0.2987 |
| Swallowing | 0.6162 | 0.4888 | 1.5893 | 0.2074 |
| Thirsty | 0.4796 | 0.7243 | 0.4385 | 0.5079 |
| Tremor | -10.8531 | 272.4 | 0.0016 | 0.9682 |
| Unintentional sweating | -0.2719 | 0.9605 | 0.0802 | 0.7771 |
| Unintentional sweating and night sweating | -0.6931 | 1.1902 | 0.3391 | 0.5603 |
| Urine | 0.2076 | 0.6349 | 0.107 | 0.7436 |
| Weakness | 1.0703 | 0.6828 | 2.4573 | 0.117 |
| White tongue coating | 0.3102 | 0.512 | 0.367 | 0.5447 |
| Yellow tongue coating | -0.7884 | 1.2631 | 0.3896 | 0.5325 |

vs.: versus

*An asterisk text indicates symptoms/signs that showed statistically significant differences between the compared groups (P < 0.05)
