# Supplementary material for: Symptom-based classification of Parkinson’s disease using retrospective electronic medical records: A STROBE-compliant cross-sectional study
Source: Medicine (Baltimore). 2026 Sep 18;105(38):e50608. doi: 10.1097/MD.0000000000050608 (PMC13593295; doi:10.1097/MD.0000000000050608)
Supplement: Supplementary file 5 [file medi-105-e50608-s005.docx]

Supplemental Table 4. Pairwise comparisons of the relative ratios for herbal medicine ingredients used for patients with Parkinson’s disease according to progressive duration

| Type of herbal medicine ingredient, n, (%) |  | Within 3 years | 3 to 7 years | Over 7 years |
| --- | --- | --- | --- | --- |
| Astringent medicinal |  | 104 (4.9) | 49 (5.0) | 37 (4.5) |
| Blood-activating and stasis-resolving medicinal |  | 73 (3.5) | 25 (2.6) | 32 (3.9) |
| Blood-tonifying medicinal |  | 237 (11.2) | 104 (10.7) | 94 (11.4) |
| Cough-suppressing and panting-calming medicinal |  | 52 (2.5) | 41 (4.2) | 25 (3.0) |
| Dampness-draining diuretic medicinal |  | 159 (7.5) | 63 (6.5) | 61 (7.4) |
| Dampness-resolving medicinal |  | 13 (0.6) | 17 (1.7) | 2 (0.2) |
| Digestant medicinal |  | 64 (3.0) | 34 (3.5) | 22 (2.7) |
| Heat-clearing medicinal |  | 229 (10.9) | 70 (7.2) | 77 (9.3) |
| Interior-warming medicinal |  | 72 (3.4) | 38 (3.9) | 31 (3.8) |
| Liver-pacifying and wind-extinguishing medicinal |  | 3 (0.1) | 1 (0.1) | 1 (0.1) |
| Orifice-opening medicinal |  | 38 (1.8) | 14 (1.4) | 16 (1.9) |
| Purgative medicinal |  | 5 (0.2) | 2 (0.2) | 1 (0.1) |
| Qi-regulating medicinal |  | 143 (6.8) | 90 (9.3) | 62 (7.5) |
| Qi-tonifying medicinal |  | 305 (14.5) | 166 (17.1) | 110 (13.3) |
| Tranquillizing medicinal |  | 106 (5.0) | 45 (4.6) | 54 (6.5) |
| Wind-clod dispersing medicinal |  | 25 3(12.0) | 96 (9.9) | 109 (13.2) |
| Wind-dampness-dispelling medicinal |  | 66 (3.1) | 21 (2.2) | 31 (3.8) |
| Wind-heart dispersing medicinal |  | 43 (2.0) | 20 (2.1) | 7 (0.8) |
| Yang-tonifying medicinal |  | 60 (2.8) | 40 (4.1) | 14 (1.7) |
| Yin-tonifying medicinal |  | 83 (3.9) | 36 (3.7) | 40 (4.8) |
