# Supplementary material for: Symptom-based classification of Parkinson’s disease using retrospective electronic medical records: A STROBE-compliant cross-sectional study
Source: Medicine (Baltimore). 2026 Sep 18;105(38):e50608. doi: 10.1097/MD.0000000000050608 (PMC13593295; doi:10.1097/MD.0000000000050608)
Supplement: Supplementary file 6 [file medi-105-e50608-s006.docx]

Supplemental Table 5. Pairwise comparisons of the relative ratios for herbal medicine ingredients used for patients with Parkinson’s disease by subtype

| Type of herbal medicine ingredient, n, (%) |  | Subtype 0 | Subtype 1 | Subtype 2 | Subtype 3 | Subtype 4 |
| --- | --- | --- | --- | --- | --- | --- |
| Astringent medicinal |  | 34 (4.7) | 48 (6.0) | 42 (4.3) | 14 (5.0) | 52 (4.6) |
| Blood-activating and stasis-resolving medicinal |  | 35 (4.8) | 23 (2.9) | 23 (2.4) | 3 (1.1) | 46 (4.1) |
| Blood-tonifying medicinal |  | 83 (11.4) | 93 (11.7) | 114 (11.8) | 28 (10.0) | 117 (10.3) |
| Cough-suppressing and panting-calming medicinal |  | 22 (3.0) | 22 (2.8) | 30 (3.1) | 11 (3.9) | 33 (2.9) |
| Dampness-draining diuretic medicinal |  | 53 (7.3) | 65 (8.2) | 57 (5.9) | 12 (4.3) | 96 (8.5) |
| Dampness-resolving medicinal |  | 6 (0.8) | 4 (0.5) | 9 (0.9) | 5 (1.8) | 8 (0.7) |
| Digestant medicinal |  | 22 (3.0) | 22 (2.8) | 35 (3.6) | 11 (3.9) | 30 (2.6) |
| Heat-clearing medicinal |  | 56 (7.7) | 93 (11.7) | 86 (8.9) | 27 (9.6) | 114 (10.1) |
| Interior-warming medicinal |  | 29 (4.0) | 21 (2.6) | 39 (4.0) | 12 (4.3) | 40 (3.5) |
| Liver-pacifying and wind-extinguishing medicinal |  | 0 (0.0) | 2 (0.3) | 1 (0.1) | 2 (0.7) | 0 (0.0) |
| Orifice-opening medicinal |  | 8 (1.1) | 17 (2.1) | 22 (2.3) | 7 (2.5) | 14 (1.2) |
| Purgative medicinal |  | 2 (0.3) | 1 (0.1) | 0 (0.0) | 1 (0.4) | 4 (0.4) |
| Qi-regulating medicinal |  | 48 (6.6) | 42 (5.3) | 94 (9.7) | 22 (7.8) | 89 (7.9) |
| Qi-tonifying medicinal |  | 130 (17.9) | 103 (13.0) | 14 7(15.2) | 47 (16.7) | 154 (13.6) |
| Tranquillizing medicinal |  | 27 (3.7) | 46 (5.8) | 65 (6.7) | 21 (7.5) | 46 (4.1) |
| Wind-clod dispersing medicinal |  | 87 (12.0) | 94 (11.8) | 99 (10.2) | 24 (8.5) | 154 (13.6) |
| Wind-dampness-dispelling medicinal |  | 20 (2.8) | 27 (3.4) | 24 (2.5) | 3 (1.1) | 44 (3.9) |
| Wind-heart dispersing medicinal |  | 11 (1.5) | 15 (1.9) | 14 (1.4) | 7 (2.5) | 23 (2.0) |
| Yang-tonifying medicinal |  | 26 (3.6) | 21 (2.6) | 21 (2.2) | 12 (4.3) | 34 (3.0) |
| Yin-tonifying medicinal |  | 28 (3.9) | 36 (4.5) | 48 (4.9) | 12 (4.3) | 35 (3.1) |
